# Isolation, Biochemical and Genomic Characterization of Glyphosate Tolerant Bacteria to Perform Microbe-Assisted Phytoremediation

**DOI:** 10.3389/fmicb.2020.598507

**Published:** 2021-01-14

**Authors:** Francisco Massot, Panagiotis Gkorezis, Jonathan Van Hamme, Damian Marino, Bojana Spirovic Trifunovic, Gorica Vukovic, Jan d’Haen, Isabel Pintelon, Ana María Giulietti, Luciano Merini, Jaco Vangronsveld, Sofie Thijs

**Affiliations:** ^1^Cátedra de Biotecnología, Departamento de Microbiología, Inmunología, Biotecnología y Genética, Facultad de Farmacia y Bioquímica, Universidad de Buenos Aires, Junín, Argentina; ^2^Instituto de Nanobiotecnología (NANOBIOTEC), CONICET-Universidad de Buenos Aires, Junín, Argentina; ^3^Environmental Biology, Centre for Environmental Sciences, Hasselt University, Diepenbeek, Belgium; ^4^Department of Biological Sciences, Thompson Rivers University, Kamloops, BC, Canada; ^5^Centro de Investigaciones del Medio Ambiente, Facultad de Ciencias Exactas, Universidad Nacional de la Plata (UNLP), La Plata, Argentina; ^6^Department of Phytomedicine, Faculty of Agriculture, University of Belgrade, Belgrade, Serbia; ^7^Institute for Materials Research (IMO-IMEC), Hasselt University, Diepenbeek, Belgium; ^8^Laboratory of Cell Biology and Histology, University of Antwerp, Antwerp, Belgium; ^9^EEA Anguil INTA-CONICET, Anguil, Argentina; ^10^Department of Plant Physiology and Biophysics, Faculty of Biology and Biotechnology, Maria Curie-Skłodowska University, Lublin, Poland

**Keywords:** glyphosate, microbe-assisted phytoremediation, EPSP synthase, glyphosate tolerance, glyphosate degradation, microcosm, plant-bacteria interaction, *phn* operon

## Abstract

The large-scale use of the herbicide glyphosate leads to growing ecotoxicological and human health concerns. Microbe-assisted phytoremediation arises as a good option to remove, contain, or degrade glyphosate from soils and waterbodies, and thus avoid further spreading to non-target areas. To achieve this, availability of plant-colonizing, glyphosate-tolerant and -degrading strains is required and at the same time, it must be linked to plant-microorganism interaction studies focusing on a substantive ability to colonize the roots and degrade or transform the herbicide. In this work, we isolated bacteria from a chronically glyphosate-exposed site in Argentina, evaluated their glyphosate tolerance using the minimum inhibitory concentration assay, their *in vitro* degradation potential, their plant growth-promotion traits, and performed whole genome sequencing to gain insight into the application of a phytoremediation strategy to remediate glyphosate contaminated agronomic soils. Twenty-four soil and root-associated bacterial strains were isolated. Sixteen could grow using glyphosate as the sole source of phosphorous. As shown in MIC assay, some strains tolerated up to 10000 mg kg^–1^ of glyphosate. Most of them also demonstrated a diverse spectrum of *in vitro* plant growth-promotion traits, confirmed in their genome sequences. Two representative isolates were studied for their root colonization. An isolate of *Ochrobactrum haematophilum* exhibited different colonization patterns in the rhizoplane compared to an isolate of *Rhizobium* sp. Both strains were able to metabolize almost 50% of the original glyphosate concentration of 50 mg l^–1^ in 9 days. In a microcosms experiment with *Lotus corniculatus* L, *O. haematophilum* performed better than *Rhizobium*, with 97% of glyphosate transformed after 20 days. The results suggest that *L. corniculatus* in combination with to *O. haematophilum* can be adopted for phytoremediation of glyphosate on agricultural soils. An effective strategy is presented of linking the experimental data from the isolation of tolerant bacteria with performing plant-bacteria interaction tests to demonstrate positive effects on the removal of glyphosate from soils.

## Introduction

Glyphosate (N-phosphonomethyl glycine) is a broad-spectrum systemic herbicide, generally known as the active compound of the commercial product Roundup. It has been widely used in agriculture since the mid-seventies. The compound has been classified as positively associated to carcinogenicity (Guyton et al., 2015), and due to its worldwide use of about 600–750 thousand tons per year (Maggi et al., 2020), concerns have been raised about its environmental fate and the risks it poses for human health. Therefore, in countries whose agricultural economy strongly depend on the use of glyphosate-associated crops such as Canada, United States, Brazil, and Argentina (Struger et al., 2015; Ronco et al., 2016; Fernandes et al., 2019; Hébert et al., 2019), the herbicide represents a major environmental and health concern.

To alleviate the effects of the non-target application as well as the off-site runoff, *in situ* degradation or transformation of glyphosate should be boosted. To this end, much attention has been directed toward exploiting plant-microbe interactions, known as microbe assisted phytoremediation, to remediate pesticides polluted soils (Glick, 2003; Kuiper et al., 2004; Vangronsveld et al., 2009).

Several studies have described the plant-associated and bulk soil bacteria from chronically exposed agricultural lands and tested them for their glyphosate degradation potential and tolerance (Zhan et al., 2018). The glyphosate degradation in bacteria occurs by two different metabolic pathways which are the AMPA (aminomethylphosphonic acid) pathway and the sarcosine pathway. The first one involves the action of an oxidoreductase (glyphosate oxidoreductase or GOX) or also a glycine oxidase, yielding AMPA and glyoxylate as degradation products. The second one involves specific phosphonates, C-P lyases, yielding sarcosine and inorganic phosphate as degradation products (Huang et al., 2016). On the other hand, bacteria tolerance to glyphosate is related to the enzyme 5-enolpyruvylshikimate-3-phosphate synthase (EPSPS, EC2.5.1.19), its only molecular target. Based on the inherent sensitivity to glyphosate the EPSPS has been divided into two types or “Classes.” Class I enzymes are those present in plants and in many Gram-negative bacteria and represent the most sensitive molecules. This class is generally represented by the EPSPS of *Escherichia coli*. Single point mutations, such as the P101S substitution (*Salmonella typhimurium*) or G96A (*Klebsiella pneumoniae*), were described for conferring herbicide tolerance. Class II enzymes can be found in tolerant bacteria, such as *Agrobacterium* sp. CP4, and they exhibit a high catalytic efficiency, even when exposed to high glyphosate concentrations. These enzymes also show important differences in the peptide sequence when compared to Class I enzymes (Pollegioni et al., 2011). In spite of contributing to what we know about these mechanisms of tolerance and degradation, publications usually focus on biomass production for bioremediation experiments, and not on its phytoremediation potential, by means of plant-bacteria interaction tests and xenobiotic dissipation.

To increase the success of phytoremediation, inoculated bacteria need to be rhizocompetent (Kuiper et al., 2004; Gerhardt et al., 2017), i.e., able to colonize and associate with the host plant in the root zone. Only few studies address or test the plant colonization potential of inoculated strains prior to bioaugmentation, and none of them referring to glyphosate phytoremediation. This ability is, however, critical to the optimization process.

In order to successfully design a field-scale phytoremediation strategy to remediate glyphosate contaminated agronomic soils, we hypothesize that it is possible to have access to plant-colonizing, glyphosate-tolerant and degrading strains and at the same time, linking them to plant-microorganism interaction studies focusing on their ability to re-colonize the roots and degrade or transform the herbicide.

As a starting point, *Lotus corniculatus* L. (birdsfoot trefoil) was previously selected and described as a glyphosate tolerant plant, suitable to grow in soils containing the highest concentrations of this herbicide that are found in agricultural fields (Massot et al., 2016). *Lotus* species and *L. corniculatus* in particular, have a worldwide distribution due to their introduction in non-native areas for use as highly productive crops in pasture systems in a diverse range of landscapes, including those of glyphosate-associated crops (Escaray et al., 2011).

To confirm our assumption, enrichment cultures containing glyphosate as the sole source of phosphorus were set up. The isolated and purified strains were subsequently biochemically characterized and the genomes of the best overall performing strains were sequenced and screened for traits related to glyphosate tolerance and glyphosate degradation, and also traits important to ensure the safety and traceability of the strains in the field (absence of plant pathogenicity clusters). To provide additional support for the selection of the most suitable strains for microbe-assisted phytoremediation, root colonization was investigated by confocal fluorescence and scanning electron microscopy followed by microcosm studies using field relevant concentrations of glyphosate.

This paper describes the whole process from the isolation of sixteen glyphosate tolerant bacterial strains from *Lotus* pastureland plots and characterization of their plant growth promotion potential, glyphosate tolerance and root colonization, to the assessment of the glyphosate degradation abilities of the two best performing ones (*Ochrobactrum haematophilum* P6BS-III and *Rhizobium* sp. P44RR-XXIV) in a microcosms assay. In addition, the whole genome sequencing underlines the novelty of the studied strains.

## Materials and Methods

### Sampling Site and Bacterial Isolation

All samples were taking in August 2014 at the Manantiales Experimental Farm of the National Institute of Agricultural Technology (INTA), in Chascomús county, Argentina (35°34′30″S 58°00′32″W). Pastureland bulk soil and rhizosphere-associated soil samples were collected from four different agricultural plots where a long-term experiment was carried out with glyphosate, to promote *Lotus spp.* as legume pastures. A high dose of glyphosate (approximately 3.5 L ha^–1^) had been applied twice a year during the last 10 years. A total of nine samples per plot were collected. Each of them with approximately 30 cm of diameter and 30 cm deep, comprising the 0 and A soil horizons and including at least one *Lotus* specimen.

In the laboratory, *Lotus* plants were carefully removed from the soil. Subsequently, they were gently shaken to remove the soil weakly attached to the roots and their aerial parts were removed. Approximately 4 g of roots were placed in flasks containing 50 ml of a 9 mg ml^–1^ NaCl and Tween 80 0.01 v/v solution, and shaken at 200 rpm for 30 min. Finally, roots were removed from the flasks, and the suspension was named as “rhizosphere.” Once plants were removed from the samples, the remaining soil was sieved (mesh size 2.0 mm) and named as “pastureland bulk soil.” A physicochemical characterization of these soils was conducted (Supplementary Table 1).

Either rhizosphere or bulk soil were used for enrichment cultures in basal salt-glyphosate medium broth (MSB-Gly). The composition of the medium was (per liter): Glucose, 2.0 g; (NH_4_)_2_SO_4_, 0.5 g; MgSO_4_.7H_2_O, 0.5 g; NaHCO_3_, 0.175; FeCl_3_.H_2_O, 10 mg; CaCl_2_.H_2_O, 10 mg; MnCl_2_, 0.1 mg; ZnSO_4_, 0.01 mg; glyphosate, 500 mg as the sole source of phosphorous (pH 6.8) (Radosevich et al., 1995). During the first three subculture rounds, cycloheximide (100 mg L^–1^) was added to avoid fungal growth. A total of six sequential subcultures were performed for each sample.

Isolation of single colonies was done by plating the cultures in solid MSB-Gly (15 g L^–1^ agar) and incubation at 25°C for 5 days. Colony purity was verified by means of streaking on different rich agar media. The conservation of the pure colonies was performed using two different techniques starting from MSB-Glyphosate liquid cultures: resuspended pellets in MSB solution and 40% glycerol, and stored in cryotubes at −80°C; and resuspended pellets in a 10% milk powder solution and then stored in cryotubes, frozen at −80°C and later lyophilized.

### DNA Isolation and Phylogenetic Analysis

Cultures of the pure strains were grown in MSB-Gly broth until reaching the stationary phase. Then 4 ml of each culture were centrifuged, and the pellet was re-suspended in 250 μl TEG (Tris, 10 mM-EDTA, 1 mM-Glucose, 50 mM) buffer (pH:8), supplemented with 5 μl lysozyme (300 mg ml^–1^), 5 μl of RNase (20 mg ml^–1^) and 20 μl of protease (7500 U ml^–1^) and incubated at 37°C for 30 min. Genomic DNA was isolated by using the Highway DNA PuriPrep-S Kit (Inbio Highway, Tandil, Argentina) according to the manufacturer’s instructions. Amplification of the 16S rRNA gene was performed by using the universal bacterial primers 27F (5′-AGAGTTTGATCMTGGCTCAG-3′) and 1492R (5′-TACGGYTACCTTGTTACGACTT-3′), T-Free DNA Taq polymerase and buffer (Inbio Highway, Tandil, Argentina), and the following PCR conditions: 3 min at 94°C, 35 cycles of 30 s at 94°C, 60 s at 55°C, and 120 s at 72°C, followed by a final extension step of 10 min at 72°C. PCR products were purified with the Highway DNA PuriPrep-GP kit (Inbio Highway, Tandil, Argentina). The amplification products were sequenced at the Genomic Research Unit of the Institute of Biotechnology CNIA-INTA (Argentina) using an automatic capillary sequencer model ABI3130XL (Applied Biosystems, Waltham, MA, United States).

The most probable taxonomic affiliation was obtained through the comparison of the obtained partial 16S rRNA gene sequences (a mean of 1350 base pairs, representing approximately 95% of the gene coverage) versus homologous sequences of prokaryote strains using the EzTaxon server of the EzBioCloud (Kim et al., 2007; Yoon et al., 2017) database and the Ribosomal Database Project server (RDP) (Cole et al., 2014).

### *In vitro* Plant Growth Promotion (PGP) Potential

To assess the *in vitro* PGP abilities of the isolated strains, a set of seven different tests were performed. The 1-aminocyclopropane-1-carboxylate (ACC) deaminase activity was estimated according to Belimov et al. (2005). A qualitative assay of Indoleacetic Acid production (IAA) was performed according to Patten and Glick (2002). The Organic Acids production was investigated following the method suggested by Cunningham and Kuiack (1992). The production of acetoin was studied using the Voges–Proskauer test, according to modifications made by Romick and Fleming (1998). The Inorganic Phosphate solubilization assay was performed following the protocol described by Nautiyal (1999). The phytate mineralizing capacity was studied according to Jorquera et al. (2008). The qualitative assessment of siderophores production was based on the method described by Schwyn and Neilands (1987).

### Glyphosate Minimal Inhibitory Concentration (MIC)

In order to assess the maximum concentration of glyphosate that each strain can tolerate, the MIC method proposed by Cockerill et al. (2012) was adopted (2012). Petri dishes with solid MSB-Gly medium containing increasing concentrations of glyphosate (>99%, Sigma-Aldrich, Buenos Aires, Argentina) were prepared (0, 100, 500, 1000, 2000, 3000; 5000, 7500, and 10000 mg glyphosate kg^–1^). To prepare the inoculum, strains were cultured in MSB-Gly medium until they reached cell densities between 1 × 10^8^ CFU ml^–1^ to 2 × 10^8^ CFU ml^–1^. Then, 2 μl of cell suspensions with an estimated total number of 1.10^4^ CFU were dropped on the agar surface. Five replicates of each strain were performed for each concentration. Plates were incubated at 25°C for 6 days and the MIC end points were determined as the concentration where the growth of the colonies was inhibited with more than 50% compared to the control (MSB without glyphosate). *Escherichia coli* DH5α (Taylor et al., 1993) (an EPSPS Class I carrier strain) was selected as a negative control. *E. coli* EPSPS is naturally sensitive, and no tolerance and/or degradation mechanisms have been reported.

### Whole Genome Sequencing and Analyses

High molecular weight genomic DNA was extracted from the cultures in the early exponential growth phase. The cells were lysed using an enzymatic lysis buffer (20 mM Tris, 2 mM EDTA, 1.2% Triton X-100, 18 mg ml^–1^ lysozyme, pH 8.0), for 30 min at 37°C. Then, the DNA was isolated using the DNeasy Blood and Tissue kit (Qiagen, Venlo, Netherlands) according to the manufacturer’s instructions. DNA concentrations were determined using the Qubit dsDNA HS Assay Kit (Thermo Fisher Scientific, Merelbeke, Belgium) and the purity using the UV-Vis NanoDrop 1000 (Thermo Fisher Scientific) spectrophotometer. The HWM DNA was subjected to the Nextera DNA Flex library preparation kit (Illumina, Eindhoven, Netherlands) according to manufacturer’s instructions. Subsequently, the indexed WGS products were sequenced on an Illumina NovaSeq 6000 system (Macrogen, Amsterdam, Netherlands).

Genome assembly was performed using the SPAdes algorithm, version 3.8.2 (Bankevich et al., 2012) (uniform coverage mode; k-mers 21, 33, 55, 77, 99). The Mauve software (Rissman et al., 2009) was used to reorder the contigs. CheckM was run to verify genome completeness and contamination (Parks et al., 2015), and a MultiQC report was generated based on the QUAST assembly statistics (Gurevich et al., 2013; Ewels et al., 2016). Genome annotation was performed using the Rapid Annotation using Subsystem Technology (RAST) annotation system (Aziz et al., 2008; Overbeek et al., 2014), NCBI Prokaryotic Genome Automatic Annotation Pipeline (PGAP) (Tatusova et al., 2016), and the MicroScope platform using the Magnifying Genomes tool (MaGe) (Vallenet et al., 2006). The Clusters of Orthologous Genes (COG) and the reconstruction of the metabolic pathways were performed using the MaGe KEGG, MetaCyc, and BioCyc tools (Caspi et al., 2016). The metabolic pathways of interest were found in this manner. For each query, homologous genes were defined according to a cut-off *e*-value of <0.0001, >20% of query coverage, and >50% sequence similarity. Genes in internal clusters were assigned using CD-HIT Suite v4.8.1 (Huang et al., 2010), genes with function prediction were found using EggNog 4.5.1 (Huerta-Cepas et al., 2016), genes with a Pfam domain were assigned using the HMMER server (Potter et al., 2018), genes with signal peptides were assigned using SignalP 5.0 server (Almagro Armenteros et al., 2019), and genes with transmembrane helices were predicted using TMHMM Server v. 2.0 (Krogh et al., 2001).

The JSpecies web server (Richter et al., 2015) and the Genome-to-genome distance calculator (Meier-Kolthoff et al., 2013) were used to select the closest genome to the strains under study.

### Glyphosate Biotransformation in Culture Medium

*Ochrobactrum haematophilum* P6BS-III and *Rhizobium* sp. P44RR-XXIV strains were aerobically grown in MSB-Gly broth (50 mg l^–1^ glyphosate) at 25°C and 200 rpm. In total, six independent cultures were made of each microorganism and the optical density (O.D.) at 600 nm, the CFU ml^–1^ count and measuring the remaining glyphosate concentration in the culture medium were considered. For the latter, triplicate supernatant samples were collected at initial time (0 h) and at ending time (216 h) and glyphosate was measured by liquid chromatography tandem-mass spectrometry (LC-MS/MS). Briefly, 100 μl of the sample was transferred to a centrifuge tube of 15.0 ml with 9.0 ml of water and 1.0 ml of 0.1 M KOH, and vortexed for 1 min. Subsequently, 1.0 ml of the mixture was transferred to a new 15.0 ml plastic tube, adding 1.0 ml of H_3_BO_3_ buffer (pH 9) and 0.5 ml of FMOC solution (10 mM Fluorenyl-methyl-oxy-carbonyl chloride in acetonitrile) to perform the derivatization reaction overnight. The derivatization reaction was stopped by adding 100 μl of 2% H_3_PO_4_ and 100 μl of 0.1 M EDTA. The samples were analyzed using an Agilent 1260 Infinity HPLC system (Agilent Technologies, Santa Clara, CA, United States) coupled with a mass spectrometer 6460 Triple Quad (Agilent Technologies, Santa Clara, CA, United States) equipped with an electrospray ionization interface set up in negative polarity. For the chromatographic separation, an Agilent Poroshell 120 EC-C18 (3.0 mm × 50 mm, 2.7 μm) column was used. The mobile phase consisted of Solution A: 90% 5 mM NH_4_Ac in H_2_O / 10% 5 mM NH_4_Ac in methanol; Solution B: 90% 5 mM NH_4_Ac in methanol / 10% 5 mM NH_4_Ac in H_2_O. To perform the chromatographic separation, the gradient was set up as follows: start with 30% B; and hold for 5 min; from 5 to 15 min, increase to 90% B, with a flow rate 0.3 ml min^–1^. For data processing MassHunter Workstation Software, version B.06.00 Agilent Technologies was used. Data were analyzed using one-way analysis of variance. The difference between the start and the end time was considered significant at *p* < 0.05. The software used for the statistical analysis was GraphPad Prism Statistics version 5.01.

### Root Colonization of *Lotus corniculatus* by GFP- and m-Cherry Labeled Strains

For the confocal microscopy experiments, the pMP4655 or pMP7604 plasmids carrying either the genes for green fluorescent protein (GFP) and tetracycline resistance (Bloemberg et al., 2000) or the mCherry gene and tetracycline resistance (Lagendijk et al., 2010), were transferred by conjugation to *Rhizobium* sp. P44RR-XXIV and *Ochrobactrum* sp. P6BS-III, respectively. These procedures involved the use of three strains: the donor strain, *E. coli* DH5α carrying either the plasmid pMP4655 with the gene that codes for green fluorescent protein (egfp) or the plasmid pMP7604 with a gene that codes for mCherry; the helper strain, *E. coli* DH5α carrying the plasmid pRK2013; and the recipient strains, P6BS-III or P44RR-XXIV, according to the conjugation. Selective media containing tetracycline (20 or 15 μg ml^–1^) and glyphosate (500 mg l^–1^) were used. The success of conjugation was verified by observing the fluorescence in selective media, BOX-PCR (Weyens et al., 2009; Supplementary Figure 1) and sequencing of the 16S ribosomal rRNA gene in the fluorescent strain. The stability of the transconjugants was tested by subculturing and microscopy.

Inoculations of *L. corniculatus* seedlings with fluorescent strains were performed in square plates with 120 mm sides. Gamborg B5 culture medium (Gamborg et al., 1968) was used for growing *L. corniculatus* in the plates. The bacterial strains were homogeneously distributed on the agar in a concentration of approximately 1 × 10^5^ CFU ml^–1^. The seedlings were superficially sterilized beforehand and put on the upper part of the medium (Supplementary Figure 2). Subsequently, plates were placed vertically and incubated in a growth chamber (12 h of photoperiod, PAR of 170 μmol m^–2^ s^–1^, 65% of relative humidity and day/night temperatures of 22°C/18°C). Five replicates per strain were made, each one containing five seedlings. After 7–10 days, seedlings were removed from the plates, thoroughly washed with sterile Phosphate Buffered Saline (PBS, 137 mM NaCl, 2.7 mM KCl, 10 mM Na_2_HPO_4_, 1.8 mM KH_2_PO4, pH: 7.4), placed in new plates with fresh medium and transported for analysis. Before analysis, roots were washed once again with sterile PBS solution and then placed on a glass slide to obtain images under an Ultra VIEW VoX microscope (Perkin Elmer, Zaventem, Belgium).

Scanning electron microscopy (SEM) images were also made. Fixation of inoculated roots was done in 4% glutaraldehyde in 1× PBS overnight. Afterward, samples were dehydrated using ethanol in an increasing gradient. Finally, the samples were frozen in tert-butyl ethanol at −20°C and sublimated later. Images were taken in a Quanta 200 FEG-SEM apparatus (FEI Company, Eindhoven, Netherlands).

### Microcosm Experiment to Assess Glyphosate Removal From Soil

A microcosm assay was conducted to assess the glyphosate removal efficiency of two different plant-microbe associations: *L. corniculatus* – *Ochrobactrum haematophilum* P6BS-III and *L. corniculatus* – *Rhizobium* sp. P44RR-XXIV. To set up the microcosms, 200 g of non-polluted agricultural plot number 4 (Supplementary Table 1) were placed in 360 ml flasks and then subjected to three autoclave cycles for 40 min every 3 days. Final soil moisture was adjusted to approximately 85% of the field capacity (0.46 g H_2_O g^–1^ dry soil).

In parallel, seeds of *L. corniculatus* were surface sterilized and germinated in Murashige–Skoog medium (Murashige and Skoog, 1962). Seedlings with roots of approximately 1 cm were selected for the experiment.

*Ochrobactrum haematophilum* P6BS-III and *Rhizobium* sp. P44RR-XXIV were grown in BSM-Gly at 200 rpm agitation and 25°C until the late exponential stage. Then, the cell culture was centrifuged at 3000 rpm and the pellet was initially resuspended in sterile saline solution (9 mg ml^–1^ NaCl) to reach a concentration of approximately 1 × 10^8^ CFU ml^–1^. A concentrated inoculation carrier solution was prepared according to the protocol mentioned by Sridhar et al. (2004) and mixed with the initial resuspended cell solution to reach a final estimated cell density of 1 × 10^7^ CFU ml^–1^.

Seedlings were transplanted to the microcosm and grown until they reached the seventh true leaf (30 days). Then, commercial glyphosate (Roundup UltraMax^®^, Monsanto 74.7% ammonium salt) was spiked into the soil to a final concentration of 5.0 mg kg^–1^ in all experimental units. Glyphosate concentration was measured 20 days after application. The experimental units were watered once per week with 5 mL of sterile saline solution throughout the experiment.

For glyphosate and AMPA (aminomethylphosphonic acid) analysis in soils, 5 g of sample was placed in a 50 ml polypropylene tube and 100 μl of a 5 mg l^–1^ solution of ^13^C-^15^N-Gly (Sigma-Aldrich International GmbH) were added to each sample as an internal standard. The samples were extracted with 25 ml of a 100 mM solution of K_2_HPO_4_ adjusted to pH = 9 with 100 mM Na_4_B_2_O_7_. The extraction was carried out by sonication at 40°C for 15 min, then centrifuged and 2 ml of the supernatant was used for precolumn derivatization (Lupi et al., 2019). Analytes in a 15 ml polypropylene tube were derivatized with a solution of FMOC-CL (9-fluorenylmethyl chloroformate; 1 mg ml^–1^ in acetonitrile) in a sample:derivatizer ratio 1:1 overnight. Subsequently, the mixture was treated with 6 ml of dichloromethane, centrifuged, and the supernatant was filtered by 0.45 μm. For chemical analysis, a liquid chromatograph Aliance 2495 (Waters^®^ Corporation, Milford, MA, United States) was used, coupled to a Quattro Premier XE tandem mass detector (Waters), with a positive mode ESI source using Nitrogen as drying gas at 410°C and Argon as collision gas at 0.35 ml min^–1^. For separation, a C18 X-SELECT column (Waters) of 75 mm × 4.6 ID was used at a flow rate of 0.5 ml min^–1^ with a water:methanol gradient, both added with 5 mM NH_4_Ac. For each analyte two mass transitions were applied, one of quantification and one of confirmation (Primost et al., 2017).

Pastureland bulk soils devoid of any roots were also inoculated, reaching an estimated final concentration of 1 × 10^7^ CFU g^–1^. Non-inoculated experimental units where spiked with the sterile carrier solution.

Data were analyzed using one-way analysis of variance and *post hoc* comparisons were performed with Dunnett significant difference tests. The difference was considered significant at *p* < 0.05. The software used for the statistical analysis was GraphPad Prism Statistics version 5.01.

## Results

### Identification and Biochemical Characterization of Glyphosate Tolerant Bacteria

Twenty-four strains were isolated from rhizosphere and pastureland bulk soil samples on solid medium, then preserved, and their survival was checked after 3 months of conservation at −80°C and freeze-drying. Sixteen colonies were able to re-grow showing a similar growth rate to that before storage (Table 1). From a total of sixteen pure strains identified using the 16S rRNA gene, eleven belonged to the genus *Rhizobium*, three to the genus *Ochrobactrum*, one to *Phyllobacterium* and one to *Pedobacter* (Table 1). Among all the *Rhizobium* species isolated, five of them have as the closest neighbor the *Rhizobium vallis* CCBAU 65647 strain type, and three of them *Rhizobium miluonense* HAMBI 2971 and CCBAU 41251.

**TABLE 1:** Origin, identification, *in vitro* plant growth promotion abilities and glyphosate tolerance of microorganisms associated to *Lotus* spp. roots.

**Source**	**Strain**	**Closest related neighbor (type strain)**	***In vitro* plant growth promotion abilities**							**Glyphosate MIC (mg Kg**^–^**^1^)**
			
			**ACC-deaminase**	**IAA**	**Acetoin**	**Ca_3_(PO_4_)_2_ Solubilization**	**Phytate mineralization**	**Organic Acids production**	**Siderophores production**	
***Lotus* spp.**	P8RR - IV	*Rhizobium vallis* CCBAU 65647	−	+	−	+	++	−	−	500
**rhizosphere**	P4RR - V	*Rhizobium mayense* CCGE526	+	−	−	+	++	−	+	7,500
	P12RR - VI	*Rhizobium vallis* CCBAU 65647	−	+	−	+++	+++	−	−	5,000
	P16RR - IX	*Rhizobium vallis* CCBAU 65647	−	+	+	+	+	−	+	10,000
	P20RR - XI	*Rhizobium vallis* CCBAU 65647	−	+	−	+	+	−	−	7,500
	P20RR - XII	*Ochrobactrum anthropi ATCC 49188*	+	+	−	++	+	+	+	10,000
	P28RR - XV	*Rhizobium miluonense HAMBI 2971*	+	−	+	+	++	−	+	7,500
	P32RR - XVIII	*Rhizobium vallis* CCBAU 65647	−	+	−	+	+	−	−	3,000
	P40RR - XXII	*Rhizobium miluonense* HAMBI 2971	+	+	−	+	+	−	+	7,500
	P44RR - XXIV	*Rhizobium lusitanum* P1-7	+	+	+	−	++	+	+	10,000

**Pastureland**	P6BS - III	*Ochrobactrum haematophilum* CCUG 38531	−	+	−	++	+++	+	+	10,000
**bulk soil**	P14BS - VII	*Rhizobium miluonense* CCBAU 41251	−	−	−	+	++	−	+	7,500
	P26BS - XIV	*Pedobacter nutrimenti* J22	+	-	+	+	++	-	+	1,000
	P30BS - XVII	*Phyllobacterium myrsinacearum* IAM 13584	−	+	+	+++	+++	+	+	10,000
	P38BS - XIX	*Rhizobium freirei PRF* 81	−	+	−	−	++	+	+	7,500
	P38BS - XX	*Ochrobactrum anthropi ATCC* 49188	+	+	+	−	−	+	+	10,000

After identification of the bacterial strains, *in vitro* plant growth promotion traits were tested (Table 1). Many positive results were observed, with IAA production, phosphate solubilization (mineral and phytate form) and production of siderophores as the most important ones, regardless of the origin of the strain. In addition, strains were tested for their tolerance to glyphosate. Six of them tolerated the highest glyphosate concentration applied (10000 mg kg^–1^). Among them are all strains belonging to the genera *Ochrobactrum* and *Phyllobacterium* sp. P30BS-XVII and two strains of *Rhizobium*, P16RR-IX and P44RR-XXIV. Another six strains tolerated glyphosate concentrations up to 7500 mg kg^–1^ (all of them belonging to the genus *Rhizobium*), while the remaining strains were inhibited by lower concentrations. The sensitive *E. coli* could only grow up to a glyphosate concentration of 100 mg kg^–1^, demonstrating that the concentration chosen for the isolation and enrichment processes was appropriate to select for tolerant microorganisms.

### Genomic Characterization of Glyphosate Tolerant Bacteria

Seven of the sixteen strains were selected for whole genome sequencing to assess the genetic basis associated to glyphosate tolerance and plant-growth promotion potential following the *in vitro* tests (Table 1). According to prokaryote databases, the *Rhizobium*, *Ochrobactrum*, and *Phyllobacterium* genomes sequenced exhibit the expected genome size. Gapped identity to closest genome of the genomes ranged from 99.6 to 84.3%. The general information of the sequences is presented in Table 2. *R.* sp. P32RR-XVIII and *R.* sp. P38BS-XIX, shared less than 85% identity and *R.* sp. P44RR-XXIV a 95.1% with their closest neighbor genome deposited in GenBank, probably contributing to intra species diversity and therefore, increasing knowledge on these plant beneficial microorganisms.

**TABLE 2:** General features of the whole genome sequences.

	***Ochrobactrum* sp.**		***Rhizobium* sp.**		***Phyllobacterium* sp.**		***Rhizobium* sp.**		***Rhizobium* sp.**		***Rhizobium* sp.**		***Rhizobium* sp.**	
	**P20RR-XII**		**P28RR-XV**		** P30BS-XVII**		**P32RR-XVIII**		**P38BS-XIX**		**P40RR-XXII**		**P44RR-XXIV**	
							
**Attribute**	**Value**	**% of Total**	**Value**	**% of Total**	**Value**	**% of Total**	**Value**	**% of Total**	**Value**	**% of Total**	**Value**	**% of Total**	**Value**	**% of Total**
Genome size (bp)	4847371	100	7077829	100	5252353	100	7197937	100	7063854	100	7069808	100	7411108	100
DNA G+C (bp)	2710165	55.9	4232542	59.8	2895097	55.12	4323081	60.1	4171912	59	4230573.1	59.8	4413315	59.55
DNA scaffolds	12	100	50	100	23	100	92	100	35	100	56	100	29	100
Total genes	4946	100	7430	100	5408	100	7918	100	7275	100	7430	100	7935	100
Protein coding genes	4844	97.9	7315	98.5	5307	98.1	7786	98.3	7162	98	7317	98.5	7745	97.6
RNA genes	100	2.02	113	1.5	95	1.76	124	1.6	109	1	113	1.52	126	1.58
Pseudo genes	2	0.04	2	0.03	6	0.11	8	0.10	4	0	0	0	64	0.80
Genes in internal clusters	18	0.37	70	0.96	24	0.45	172	2.21	41	0.57	72	0.98	83	1.07
Genes with function prediction	4215	87.0	6178	84.5	4627	87.2	6303	81.0	6153	85.9	6157	84.1	6742	87.0
Genes assigned to COGs	3846	79.4	5586	76.4	4213	79.4	5602	71.9	5545	77.4	5578	76.2	6046	78.1
Genes with Pfam domains	3988	82.3	7055	96.4	5197	97.9	7410	95.2	6918	96.6	7065	96.6	7553	97.5
Genes with signal peptides	655	13.5	885	12.1	713	13.4	471	6.0	900	12.6	875	12.0	987	12.7
Genes with transmembrane helices	1240	25.6	1624	22.2	1299	24.5	1674	21.5	1624	22.7	1616	22.1	1786	23.1
Completeness		100.0		100.0		99.6		99.3		99.9		100.0		99.5
Gapped identity to closest genome		99.6		99.6		NA		84.6		84.3		99.6		95.1
Accession Number	JAANOS000000000.1		JABAIG000000000		JACGXO000000000.1		JABAIH000000000		JABAIJ000000000		JABAII000000000		MPVZ00000000.2	
BioProject	PRJNA547107		PRJNA625371		PRJNA547125		PRJNA625372		PRJNA625376		PRJNA625374		PRJNA354620	

*NA, Not available.*

The search for the nucleotide sequence of the enzyme 5-enolpyruvylshikimate-3-phosphate synthase (EPSPS, EC2.5.1.19), the enzyme inhibited by glyphosate, unveiled differences in the investigated strains. *Ochrobactrum* sp. P20RRXII, *Phyllobacterium* sp. P30BS-XVII, *Rhizobium* sp. P38BS-XIX, and *Rhizobium* sp. PP44RR-XXIV presented a single copy of the EPSPS. *Rhizobium* sp. P28RR-XV, *Rhizobium* sp. P32RR-XVIII, and *Rhizobium* sp. P40RR-XXII on the other hand, showed two copies in their genome. Among those genomes with two variants of the enzyme, *Rhizobium* sp. P28RR-XV and *Rhizobium* sp. P40RR-XXII (most likely neighbor *Rhizobium milounense*) possess the same pair of sequences.

**FIGURE 1:**
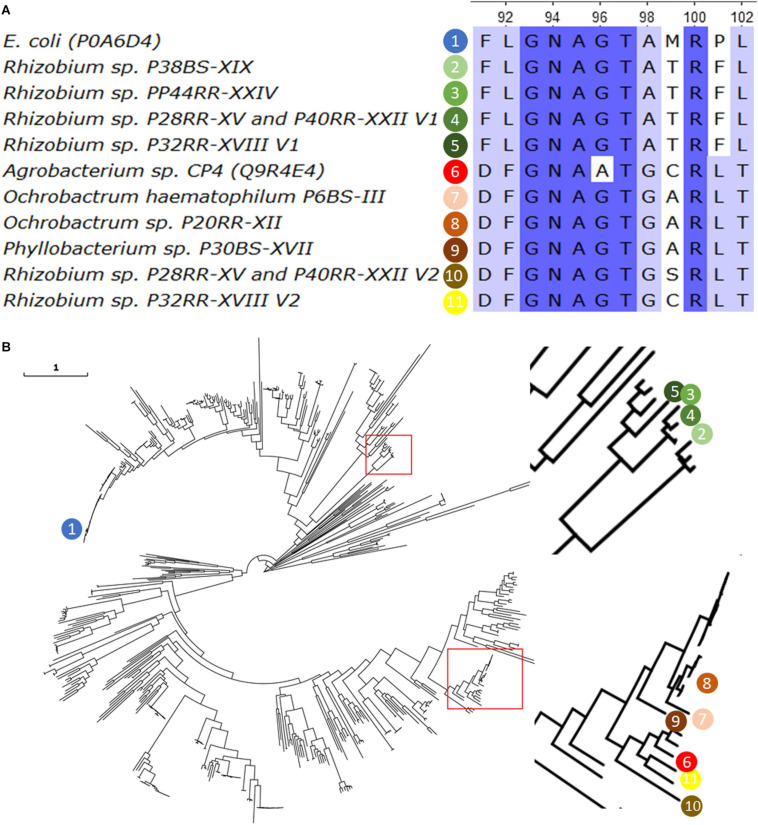
The Sequence of the 5-enolpyruvylshikimate-3-phosphate synthase (EPSPS, EC2.5.1.19) found in the fully sequenced genomes was aligned using the *ClustalW* algorithm, together with the most representative Class I (*E. coli*) and Class II (*Agrobacterium CP4*) enzymes. A fragment of twelve amino acids from position 91 to 102 (according to *E. coli* sequence) and including the 101 position is presented **(A)**. A phylogenetic tree was build using 589 EPSPS reviewed sequences from UniProt (www.unirpot.org) and those found in the investigated genomes. Sequences were aligned using ClustalO v1.2.4, and the Maximum-Likelihood phylogenetic tree were calculated using IQ-Tree v1.6.12 selecting the best substitution model based on BIC score with the option -m TEST. Confidence levels for individual branches were determined by ultrafast bootstrap approximation with 10000 replicates. The image was magnified in those branches where the sequences of interest are located, which are indicated in numbered colored spheres **(B)**. The EPSPS sequence of O. *haematophilum* P6BS-III was included in the analysis for comparative reasons.

The amino acid sequences were compared with those of *E. coli* and *Agrobacterium* sp. CP4. Figure 1A shows an alignment fragment of the highly conserved region XLGNAGTAXRXL which is critical for the attachment of phosphoenolpyruvate in Class I enzymes (Yi et al., 2016). Two different sequences can be distinguished, those similar to the typically sensitive EPSPS Class I of *E. coli* with an exception for the substitution P101F, and those similar to the naturally occurring tolerant EPSPS Class II of *Agrobacterium* sp. CP4, except for the G96A substitution associated to a complete insensitivity to glyphosate.

A phylogenetic analysis of a total of 599 EPSPS protein sequences including the 10 obtained in this work, and the *Ochrobactrum haematophilum* P6BS-III single sequence (Massot et al., 2019) was carried out. The results grouped the sequences in two clusters, one of them including the sequence of the CP4, and the other one including sequences of the *Rhizobium* genus and at a significant distance of the typically sensitive EPSPS Class I of *E. coli*, with less than approximately 40% of amino acid identity (Figure 1B).

A further analysis on the sequences revealed that all of those closely related to CP4 EPSPS, presented all the critical domains important for glyphosate tolerance and phosphoenolpyruvate binding in Class II EPSPS (Sun et al., 2005).

**FIGURE 2:**
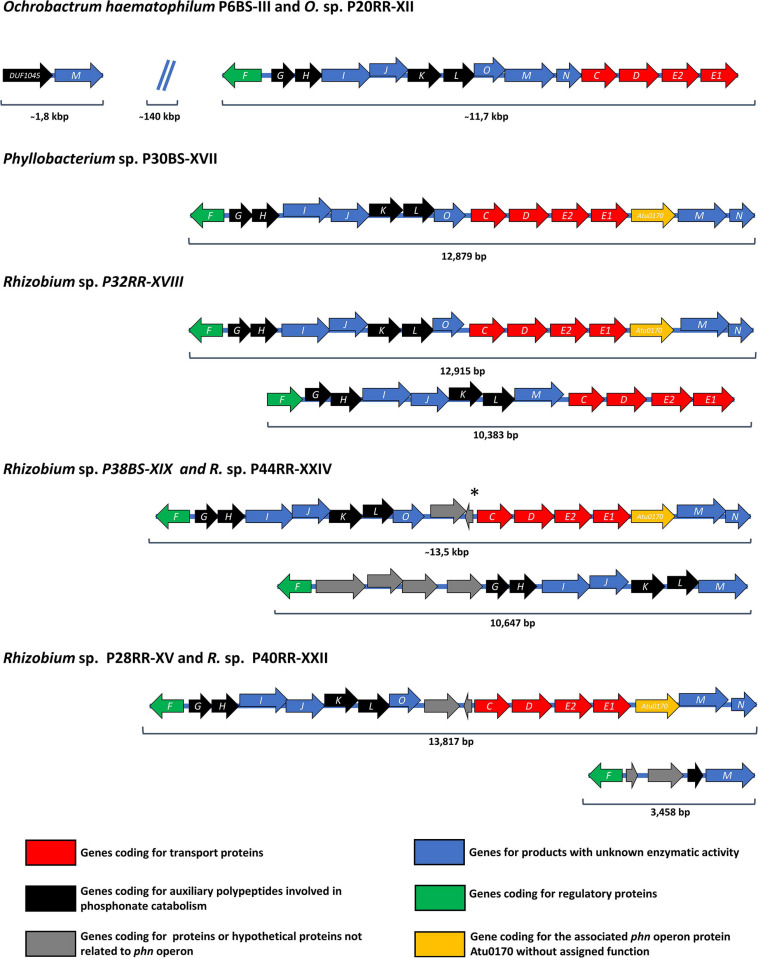
Organization of C-P lyase pathway-encoding *phn* operons in the investigated microbial strains. Open reading frames are shown as arrows indicating the direction of transcription and including the specific letter designation of the *phn* operon inside. A Displacement of an open reading frame relative to the previous open reading frame indicates an overlapping of the reading frames. At the bottom of the figure, colored squares indicate the function of each open reading frame presented above in the figure. The color nomenclature corresponds to that presented in Hove-Jensen et al. (2014). The *phn* operon organization of O. *haematophilum* P6BS-III was included in the analysis for comparative reasons. *Hypothetical open reading frame not present in *Rhizobium* sp. P38BS-XIX.

A search of the homologous sequences corresponding to the operon *phn*, a Carbon-Phosphorus Lyase responsible for the metabolization of phosphonates such as glyphosate, was also performed. *Ochrobactrum* strains and *Phyllobacterium* sp. P30BS-XVII presented a unique possible transcriptional unit, while *Rhizobium* strains presented two possible transcriptional units with the exception of *Rhizobium* sp. P28RR-XV and P40RR-XXII, which showed a 3,458 bp length transcriptional unit containing the *phnF*, *phnM*, and a fragment of *phnL* (Figure 2). The operon structure of *Ochrobactrum anthropi* as well as *Agrobacterium radiobacter* were previously reported and compared (Hove-Jensen et al., 2014). The operon organization of the genus *Phyllobacterium* was never described in detail.

All the *Rhizobium* genomes with an additional *phn* operon, presented a questionable 1-aminoalkylphosphonic acid catalytic activity, since they lack the aminoalkylphosphonate N-acetyltransferase activity provided by *phnO.* Closely related species like *Mesorhizobium loti* and *Sinorhizobium meliloti* bear plasmids containing *phn* operons but differing in structure compared to the additional operons found (Kaneko et al., 2000; Finan et al., 2001). The *Phn* operons of *Rhizobium* spp. P28RR-XV, P38BS-XIX, P40RR-XXII, and P44RR-XXIV have hypothetical genes which at first would not be related to their activity but were not previously observed in the genus.

Among all the studied strains, *Ochrobactrum* sp. P6BS-III and *Rhizobium* sp. P44RR-XXIV were selected for a more detailed characterization and plant-bacteria interaction tests. *Ochrobactrum* sp. P6BS-III, recently reported as *Ochrobactrum haematophilum* P6BS-III (Massot et al., 2019), showed various *in vitro* plant growth promotion abilities, such as production of IAA, phosphorus solubilization, and siderophores production. The *Ochrobactrum* genus was previously reported with the ability of degrading different organic compounds (Kiliç, 2009; Abraham and Silambarasan, 2016; Chudasama and Thaker, 2017). On the other hand, *Rhizobium* sp. P44RR-XXIV, identified according to its 16s rRNA gene as *Rhizobium lusitanum* (Valverde et al., 2006) showed the highest glyphosate tolerance (≥10,000 mg kg^–1^), as well as a wide range of *in vitro* PGP abilities (in particular, the ability to produce IAA, acetoin, and ACC deaminase activity).

A total of 29 contigs greater than 1000 bp, giving a consensus length of 7,408,308 bp and a mean genome coverage of 61.5 times was obtained for *Rhizobium* sp. P44RR-XXIV. The reordering of contigs was performed using the closest *Rhizobium* reference genome completely sequenced, *Rhizobium tropici* CIAT 899 (GCA_000330885.1). The P44RR-XXIV genome was also compared to two other genomes, the closest match according to the information of the 16s rRNA gene, *R. lusitanum* P7 (NZ_FMAF00000000.1), and the closest match according to results obtained using the ANIm, ANIb and GGDC analysis, *Rhizobium* sp. AC27/96 (NZ_LXKN00000000.1) (Supplementary Table 2 and Supplementary Figure 3).

Surprisingly, *Rhizobium* sp. P44RR-XXIV does not possess homologous genes related to nitrogen fixation (*nif* genes), nor NOD nodulation factors genes (*nod* genes). This may indicate that the strain is not capable of forming a symbiotic association with *Lotus* through nodule formation.

Even though this finding does not hinder the remediation strategy for which the use of these strains is intended, since P44RR-XXIV is phylogenetically close to the phytopathogen *Rhizobium rhizogenes* (Ream, 2009), an additional bioinformatic analysis was performed before performing further analyses. Strain P44RR-XXIV was then compared to the closest *Rhizobium rhizogenes* K599 (previously known as *Agrobacterium rhizogenes*), a strain that causes the “hairy root disease.” The ability to cause that disease relies on the possession of the Ri-plasmid (Hodges et al., 2006). A low synteny was observed in *R. rhizogenes* K599 with respect to the *Rhizobium* genomes in this study (Supplementary Figure 4). In order to study the presence of Ri-plasmid, queries of genes present in pRi1724 plasmid belonging to *R. rhizogenes* MAFF301724 (Moriguchi et al., 2001) were searched using BLAST. No evidence of sequences related to T-DNA, the opine synthesis “*ocp_h2*” and genes *virA*, *virH*, *virF*, and part of *virD* homologs were found. The absence of homologs of *rol* genes together with the absence of those necessary for the complete synthesis of any particular type of opine: genes *virD1*, *virD2* or *virE* genes, plus the fact that the regions that present homology correspond to different contigs within the genome of P44RR-XXIV, would indicate the absence of a functional Ri-plasmid in P44RR-XXIV.

*Rhizobium* sp. P44RR-XXIV possesses a single copy of the gene *acdS* that codes for the enzyme ACC deaminase (1-aminocyclopropane-1-carboxylate deaminase, EC 3.5.99.7). Next to the *acdS* sequence, a homolog of the *acdR* protein regulator was found in 5′ region.

The presence of genes involved in different Indoleacetic Acid (IAA) synthesis pathways was studied. Homologs of the aldehyde dehydrogenase gene, *aldA* (indole-3-pyruvate pathway), the nitrile and nitrile hydratase genes, *nit4* and *nthA-nthB* (indole-3-acetonitrile/indole-3-acetamide pathways), and a possible homolog of indoleacetamide hydrolase, *iaaH* (indole-3-acetamide pathway) were found.

There is a total of 87 genes involved in phosphorus uptake and metabolism. These are related to the high affinity phosphate transporter and the *PHO* regulon, the different phosphate metabolism pathways (including homologs of alkaline phosphatase), metabolism of polyphosphates and of alkylphosphonates (including the genes of the two *phn* operons).

Iron uptake is probably performed through several transporters, such as the ABC transporters *pitA*, *pitD*, *pitC* and the iron-B12-siderophore-hemin system. Genes related to the synthesis of siderophore Aerobactin were found.

### *Lotus corniculatus* Root Colonization

**FIGURE 3:**
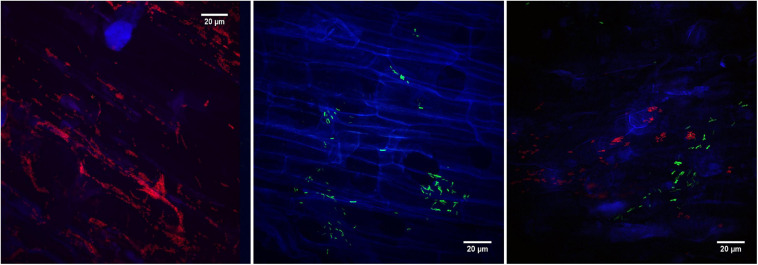
Confocal fluorescence microscopy images of *L. corniculatus* roots inoculated with the glyphosate degrading microorganisms. On the **left**, *O. haematophilum* P6BS-III m-Cherry + close to the tip of the main root. On the **middle**, *R.* sp. P44RR-XXIV GFP+. On the **right**, *L. corniculatus* roots were inoculated with *O.* sp. P6BS III mCherry+ and *R.* sp. P44RR-XXIV GFP+, where different locations in the root can be observed for each microorganism.

Using fluorescence microscopy, the two strains showing promising glyphosate metabolization abilities were tested for their potential to colonize the roots of *Lotus corniculatus*. Fluorescence was observed in the transconjugants *O. haematophilum* P6BS-III and *Rhizobium* sp. P44RR-XXIV and consequently, these strains were identified as *O. haematophilum* P6BS-III mCherry+ and *Rhizobium* sp. P44RR-XXIV GFP+. Confocal fluorescence microscopy imaging (Figure 3 and Supplementary Figure 5) showed different colonization patterns of the microorganisms over the root surface after 1 week. P6BS-III mCherry+ biofilms were observed associated in small and dense cell clusters next to the cell wall junctions. P44RR-XXIV GFP+ seemed to be more uniformly distributed and possibly associated to hairy roots, mainly occurring as individual cells, and in lower numbers than P6BS-III mCherry+.

**FIGURE 4:**
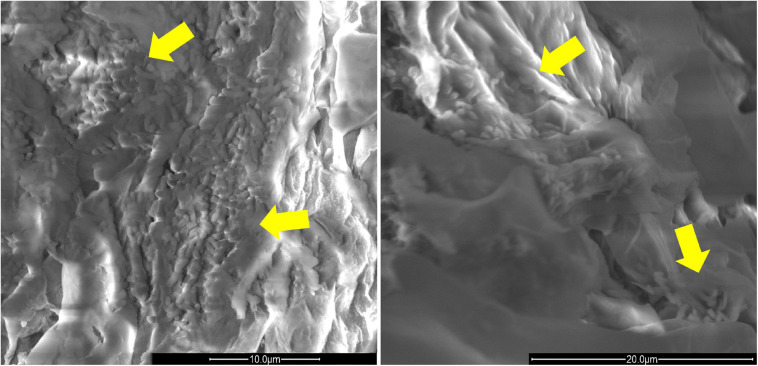
Scan electron microscopy images of *L. corniculatus* roots inoculated with *O. haematophilum* P6BS-III and *R*. sp. P44RR XXIV. Yellow arrows indicate the location of the microbial cells in the root surface. The image has been magnified 8000 times. On the **left**, *O. haematophilum* P6BS-III. On the **right**, *Rhizobium* sp. P44RR XXIV.

The presence of *Rhizobium* sp. P44RR-XXIV and *O. haematophilum* P6BS-III on the roots of *L. corniculatus* was also corroborated using scanning electron microscopy (Figure 4).

### Glyphosate Biotransformation in Liquid Cultures and Soil Microcosms Studies by Two Selected Bacteria

In order to perform bacterial cell cultures in BSM-Gly broth, two pre-cultures were made to remove any possible source of phosphorus, and thus only glyphosate as the sole source of phosphorous was left. In parallel, negative controls (BSM without glyphosate) were included.

*Ochrobactrum haematophilum* P6BS-III reached the maximum growth on day two, showing approximately 2 × 10^11^ CFU ml^–1^ in the culture medium. *Rhizobium* sp. P44RR-XXIV showed a similar growth, reaching a maximum of 2.7 × 10^10^ CFU ml^–1^. The amount of glyphosate metabolized at the end point (day 9 of cultures) was 21 mg Kg^–1^ (42% of the initial concentration) for *O. haematophilum* P6BS-III and 24.5 mg kg^–1^ (49% of the initial concentration) for *Rhizobium* sp. P44RR-XXIV. Aminomethylphosphonic acid (AMPA) was not detected in the culture medium.

Microcosms assays were conducted using the two possible plant-bacteria associations, *L. corniculatus* – *O. haematophilum* P6BS-III and *L. corniculatus* – *Rhizobium* sp. P44RR-XXIV. Additionally, glyphosate concentrations were studied in microcosms in which bacteria were spiked directly on the bulk soil to evaluate the effects of the microbes themselves.

**FIGURE 5:**
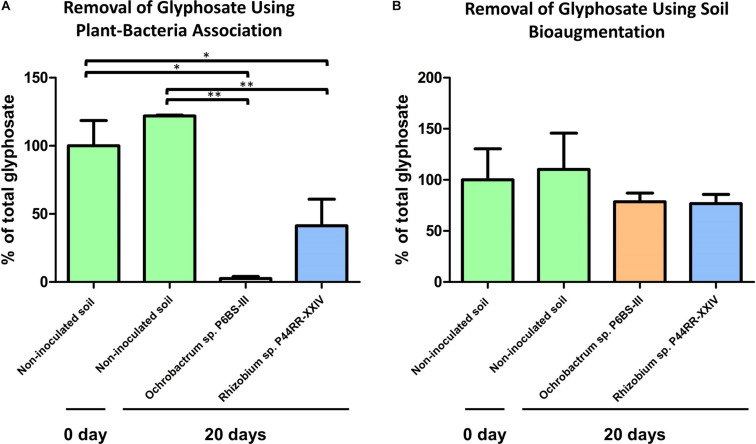
Microcosm experiments. Effect of the plant-bacteria associations on the glyphosate metabolization in soil after 20 days **(A)**. Effect of the bacteria bioaugmentation in soil after 20 days **(B)**. *P value = 0.01 to 0.05 **P value = 0.001 to 0.01.

After 20 days post treatment, plant-bacteria association microcosms showed significant decreases in the glyphosate concentration, with the *Lotus corniculatus* – *O. haematophilum* P6BS-III combination yielding the higher glyphosate decrease (a mean of 97.4% of the total glyphosate was removed, *P* value < 0.01). *L. corniculatus* – *Rhizobium* sp. P44RR-XXIV generated a 58.7% lower glyphosate concentration (*P* value < 0.05). Autoclaved soils with non-inoculated *Lotus* plants showed no significant difference in glyphosate concentration at the end of the experimental period (*P* value > 0.05). Soil microcosms inoculated only with bacteria did not show significant differences compared to non-inoculated soils (Figure 5).

## Discussion

We characterized *Lotus*-associated rhizospheric bacteria obtained from a glyphosate chronically exposed pastureland to perform a successful microcosm phytoremediation assay using a realistic agronomic dose of the herbicide. This work presents the first plant-microbe assisted phytoremediation of glyphosate in which the presence of bacteria associated to roots and an effective glyphosate biotransformation has been demonstrated.

Despite the general and widespread use of glyphosate, its phytoremediation has been poorly investigated. Jacklin et al. (2020) evaluated the effects of glyphosate in 14 plant species in constructed wetlands. The planted wetlands removed an average of 96.8% of the 0.7 mg L^–1^ glyphosate while the unvegetated wetlands (control) removed 92.2%, suggesting the relevance of the glyphosate adsorption to the soil and the need to ensure the local biodegradation. The aquatic macrophyte *Lemna minor* was used to remove a commercial formulation of glyphosate from the growth medium, achieving 8% removal at the end of the experimental period (Dosnon-Olette et al., 2011). An attempt to realize a microbe-assisted phytoremediation system was made by Kryuchkova et al. (2014), in which *Enterobacter cloacae* K7, a glyphosate degrading strain which possessed a number of associative traits, including nitrogen fixation, phosphate solubilization, and IAA synthesis, was tested in *Helianthus annuus* and *Sorghum saccharatum*. The assay was carried out in Petri dishes, and no glyphosate degradation was evaluated. Such attempts sustain the need for am appropriate evaluation of the effects of microbial inoculation on plants together with the monitoring of glyphosate concentration. In this work, *in vitro* plant growth promotion abilities tests were performed as a part of a multipurpose strategy in which the plant-microorganism association should not only provide the degradation of the xenobiotic but should also possess traits that sustain its presence in the root and eventually contribute to increase the plant biomass. A similar procedure was followed by Becerra-Castro et al. (2011, 2013), who first isolated hexachlorocyclohexane tolerant and beneficial bacteria strains from *Cytisus striatus* and later selected those with the best performance to test the dissipation of the insecticide lindane in pot experiments.

Our work evidences the isolation procedure delivered great selectivity. Almost all the obtained bacteria belonged to the order Rhizobiales, in particular, *Rhizobium* and *Ochrobactrum* genera. Rhizobiales constitute a fraction of bacteria inhabiting the roots of plants (rhizobacteria); they are promoting the growth of and alleviating the stress experienced by their host plants and present a high relative abundance in *Lotus* species (Zgadzaj et al., 2016). The association between leguminous plants and rhizobia has been proposed for phytoremediation of contaminated soils (Brígido and Glick, 2015). In the case of soils contaminated with organics, some examples can be found in which *Rhizobium* and *Ochrobactrum* genera were used in combination with members of the *Fabaceae* family. *Rhizobium meliloti* ACCC17519 in association with *Medicago sativa* was reported to reduce the soil polycyclic aromatic hydrocarbon (PAHs) concentration by 51.4% compared with unplanted control soil (Teng et al., 2011). In a study by Madariaga-Navarrete et al. (2017), *Phaseolus vulgaris* was inoculated with a *Rhizobium* sp. previously isolated from roots, to investigate the atrazine removal. The treatment was able to remove an average of 61% of atrazine 50 from soil after 35 days. An *Ochrobactrum* sp. obtained from bulk soil from coking plant, was used in association with *M. sativa* to remove PAHs. The duo was able to remove 30% of the xenobiotics after 90 days (Xu et al., 2020). *Rhizobium* and *Ochrobactrum* genera were previously reported to degrade glyphosate, but not in combination with plants (Sviridov et al., 2015; Stosiek et al., 2020).

Whole genome sequencing represents an excellent approach to study and confirm the presence of the pesticide biodegradation pathways and other relevant traits in bacteria when it is coupled with biochemical and physiological tests. For instance, a genomic and functional approach study was carried out to understand the detoxification of atrazine by *Arthrobacter* sp. C2 (Cao et al., 2021) and DDT by *Stenotrophomonas* sp. DDT-1 (Pan et al., 2016). In our work, the glyphosate metabolization through the C-P lyase pathway was investigated searching homologous gene sequences in the genome of seven strains. At least one copy of the full version of the operon was found in each strain, including the *PhnGHIJK* component essential for the catalytic activity (Kamat et al., 2013). Despite being a widely distributed operon in bacteria, C-P lyases present in many of them are not capable to degrading glyphosate (Villarreal-Chiu et al., 2012), such is the case of *E. coli* (Chen et al., 1990). Therefore, the annotation of the total of 14 polypeptides present in the *phn* operon, *phnCDEFGHIJKLMNOP*, is not sufficient to indicate microorganisms are able to metabolize glyphosate by using the sarcosine pathway. The fact that the isolation of strains was conducted using enrichment cultures in which their only source of phosphorus was glyphosate, may indicates bacteria have an active C-P lyase pathway. However, for *O. haematophilum* P6BS-III and *Rhizobium* sp. P44RR-XXIV, the metabolization of glyphosate was verified measuring it on liquid cultures. The metabolization of glyphosate through the AMPA pathway was also investigated in the genomes of *O. haematophilum* P6BS-III and *Rhizobium* sp. P44RR-XXIV. Yet, no homologous gene sequences of the Glyphosate oxidoreductase (GOX), responsible for the glyphosate decarboxylation were found. This evidence was supported by the absence of AMPA in liquid cultures. *Rhizobium* and *Ochrobactrum* species without the ability to oxidize glyphosate through the AMPA pathway were previously reported (Liu et al., 1991; Firdous et al., 2017).

The *epsps* gene was also investigated in whole genome sequences. Results showed some microorganisms possessing two complete sequences of the *epsps*, a finding that has not being reported to date. Additionally, results also supported the discovery of a new cluster of glyphosate tolerant sequences that could provide a new source of genes and proteins with properties for environmental applications. Firstly, the highly conserved region XLGNAGTAXRXL was explored since some mutations in this region have been studied extensively and can be directly associated with glyphosate tolerance. For instance, the substitution G96A can increase the glyphosate tolerance to 100 folds, while the combination of the substitutions in T97 and P101 can provide the conformational changes and lead to high catalytic efficiency and glyphosate tolerance (Pollegioni et al., 2011; Yi et al., 2016). The alignment performed delivered two groups of sequences, one of them related to Class I, and the other group related to Class II enzymes. The substitution P101F found in Class I enzymes is not among the previously P101 reported mutations and provides a variable tolerance in *Rhizobium* strains that carried them. However, it seems that belonging to this group is enough to confer a high level of tolerance (7,500 to at least, 10,000 mg Kg^–1^ of glyphosate). All strains possessing Class II related sequences showed the highest level of tolerance tested. A phylogenetic analysis was conducted to find out how similar Class I enzymes are between themselves and, at the same time, how distant the complete hypothetical peptide sequences are from the sensitive Class I sequence of *E. coli*. Our findings unveiled a potential new cluster of naturally tolerant EPSPS sequences belonging to Class I group, significantly distant from the *E. coli* sequence. Other *Rhizobium* EPSPS sequences were found conforming the clade: *Rhizobium etli* CIAT 652, *R. etli CFN 42 Rhizobium leguminosarum bv. trifolii* WSM2304. Surprisingly, strains carrying two versions of the *epsps* gene, *Rhizobium* sp. P28RR-XV, *Rhizobium* sp. P32RR-XVIII, and *Rhizobium* sp. P40RR-XXII did not show the highest tolerance to glyphosate, leading to hypothesize the absence of a functional product of the Class II gene. Future studies must endeavor to further corroborate this.

Since they showed a broad set of plant growth promotion traits, tolerated the maximum concentration of glyphosate tested and also possess a single *epsps* gene copy representative of each class, *O. haematophilum* P6BS-III and *Rhizobium* sp. P44RR-XXIV were chosen to study root colonization using fluorescence microscopy. The metabolization of glyphosate by these strains was tested in liquid cultures prior to perform the microcosms assay, demonstrating significant glyphosate metabolization after 9 days of incubation. A peculiarity of *Rhizobium* sp. P44RR-XXIV is the absence of *nif* and NOD genes, suggesting that strain P44RR-XXIV is not able to establish a symbiotic association with *Lotus* through nodule formation, but possibly through different mechanisms. This finding has already been documented in Rhizobiaceae and is of great interest for the scientific community since the ecological role that such saprophytes play in the rhizosphere remains unknown (Giraud et al., 2007; Jones et al., 2016; Okazaki et al., 2016). It has been reported that *Rhizobium etli* strains lacking *Sym* plasmids were able to successfully colonize roots (López-Guerrero et al., 2012) as it turned out for *R.* sp. P44RR-XXIV in this work.

Plant inoculation should be combined with monitoring of the bacterial survival and establishment in the rhizosphere (Errampalli et al., 1999; Ramos et al., 2013). Fluorescent tagging was used as a marker to observe the colonization and spatial distribution of the strains at the *Lotus* primary roots. This method has been previously applied in the context of microbe-assisted phytoremediation. For instance, the endophyte *Methylobacterium* sp. Cp3-mCherry was demonstrated to colonize the root cortex cells and xylem vessels of the stem of *Crotalaria pumila* under metal stress (Sánchez-López et al., 2018). In another study conducted by Pawlik et al. (2020), *Lolium perenne* was inoculated with the GFP-tagged *Rhizobium sp. 5WK* collected from an aged petroleum hydrocarbon polluted soil to confirm the endophytic nature of the strain colonization capabilities. In our work two aspects guided the search for bacteria capable of establishing in the rhizoplane of *Lotus*: (1) no relevant glyphosate degradation is accomplished in plants (Sammons and Gaines, 2014) and (2) due the low hydrophobicity of glyphosate (logK_*ow*_ < −3.2), plant roots do not take it up at a rate surpassing passive influx into the transpiration stream. Therefore, it is not surprising that the inoculated glyphosate degrading strains remain in the rhizoplane of the host. The root colonization pattern observed by confocal fluorescence microscopy was supported using SEM imaging, corroborating the long-term interaction and bacteria adhesion to plant cell wall. The combination of fluorescence microscopy and SEM to gain a reliable characterization of the colonization patterns is commonly adopted in evaluations of beneficial bacteria (Palmqvist et al., 2015; Gamez et al., 2019).

Finally, the suitability of plant bacteria associations was tested in a microcosm study by investigating the glyphosate removal in a typical agricultural soil. The dose of the herbicide was based on a commercial product application of approximately 4 kg ha^–1^ homogenously distributed in the soil, leading to a final concentration of 5 mg Kg^–1^. Glyphosate concentrations commonly found in agricultural soils are usually below 5 mg Kg^–1^ (Aparicio et al., 2013; Silva et al., 2019). Therefore, the glyphosate concentration used in the microcosm experiment is most likely the highest concentration the plant-bacteria system will be faced with in the field. Significant decreases of the glyphosate concentration were observed in the microcosms in which plant-bacteria associations were used. On the other hand, microcosms using only bacteria spiked directly on the bulk soil (bioaugmentation approach) showed no significant metabolization of glyphosate after 20 days. In a similar experiment, Ermakova et al. (2010), found that *Achromobacter* sp. Kg 16 and *Ochrobactrum anthropi* GPK were able to aerobically degrade 65.8% and 49.5% of glyphosate, respectively, in soils contaminated with approximately 60 mg Kg^–1^ of the herbicide 21 days after treatment. In that case, the degradation of glyphosate was performed by the action of the indigenous microorganisms and the degrading bacteria. In our experiment, the remotion of the indigenous microorganisms by soil sterilization proved the inability of the inoculated microorganisms to remove for themselves the xenobiotic. The results obtained in the microcosms with plant-bacteria associations illustrate the relevance of the symbiosis with plants to boost the glyphosate metabolization by bacteria. Most likely, the roots exudates of *Lotus* generate an environment with a high availability of nutrients for the heterotrophic bacteria inhabiting the rhizosphere and colonizing the root, which is increasing the microbial biomass and enhancing the metabolic activity (Segura and Ramos, 2013). Additionally, plants increase the oxygen pressure, porosity, and permeability of the soil near the roots, therefore ensuring the occurrence of aerobic reactions (Gerhardt et al., 2009). The fact that there was no significant decrease of glyphosate in the microcosm units with *L. corniculatus* without inoculation of bacteria indicates that no significant plant uptake was taking place.

## Conclusion and Perspectives

Currently, agrochemicals in general and glyphosate in particular, are amongst the major environmental concerns in the agriculture-based economies. Glyphosate and its associated products being a core technology in the prevailing agricultural model, it is unlikely that their use will decrease in the near future. This work aimed to gather the necessary knowledge to design a biotechnological tool for the microbe-assisted phytoremediation of glyphosate in agricultural soils, attempting to take the first step toward generating progressive changes that result in an improvement of the agroecosystem through the reduction of environmental liabilities.

In this study, twenty-four bacterial strains were isolated from roots and soils chronically exposed to glyphosate and sixteen of them assessed according to their glyphosate tolerance and degradation capabilities, and plant growth promotion abilities. Almost all strains belong to the order of Rhizobiales, a promising result, since there exist many reports on plant beneficial microorganisms belonging to this group. In addition, all strains comply with a tolerance level several orders of magnitude higher than the maximum reported glyphosate concentration in soils and a considerable number of *in vitro* PGP abilities. All strains have not less than three positive results in the different *in vitro* PGP traits studied, where phosphorus solubilization stands out. The acquisition of new genomic data arising from microorganisms whose genomes have not been sequenced or studied before, contributes significantly to a better comprehension of the genus. *O. haematophilum* P6BS-III and *R.* sp. P44RR-XXIV were studied for their root colonization, indicating positive adherence to *L. corniculatus* roots. *In vitro*, both strains were able to metabolize almost 50% of the original glyphosate concentration of 50 mg l^–1^ in 9 days. Lastly, in a microcosm experiment using *L. corniculatus*, *O. haematophilum* P6BS-III performed better than *R.* sp. P44RR-XXIV, with 97% of glyphosate transformed after 20-days.

This paper presents an improved proposal to existing literature, linking the experimental data from the isolation of tolerant bacteria with performing plant-bacteria interaction tests to prove positive effects on glyphosate removal from the soil. The concatenation of all the information acquired leads the authors to propose that microbe-assisted phytoremediation of glyphosate is possible starting from the bioaugmentation of a properly described microorganism and followed by the inoculation of a plant species of commercial value.

## Data Availability Statement

The datasets presented in this study can be found in online repositories. The names of the repository/repositories and accession number(s) can be found below: PRJNA547107, https://www.ncbi.nlm.nih.gov/bioproject/PRJNA547107; PRJNA 625371, https://www.ncbi.nlm.nih.gov/bioproject/PRJNA625371; PRJNA547125, https://www.ncbi.nlm.nih.gov/bioproject/PRJNA 547125; PRJNA625372, https://www.ncbi.nlm.nih.gov/bioproject/PRJNA625372; PRJNA625376, https://www.ncbi.nlm.nih.gov/bioproject/PRJNA625376; PRJNA625374, https://www.ncbi.nlm.nih.gov/bioproject/PRJNA625374; PRJNA354620, https://www.ncbi.nlm.nih.gov/bioproject/PRJNA354620.

## Author Contributions

FM wrote the manuscript and executed the main part of the experimental work and data analysis. PG assisted and advised FM during the experimental work. JV assisted ST in the genome sequencing. DM performed glyphosate measurements in microcosms studies. BT and GV performed glyphosate measurements in culture medium. Jd’H assisted FM and ST in Scan Electron Microscopy. IP assisted FM and ST in Confocal Microscopy. AG, LM, and JV experts in phyllo- and phytoremediation, critically reviewed the manuscript, and provided invaluable contributions to FM during this work. ST performed the genome sequencing of the microorganisms, performed microscopy imaging experiments with FM, and accompanied and guided FM during this work and provided a critical review to this manuscript. All authors contributed to the article and approved the submitted version.

## Conflict of Interest

The authors declare that the research was conducted in the absence of any commercial or financial relationships that could be construed as a potential conflict of interest.
