# Supplementary material for: Isolation, Biochemical and Genomic Characterization of Glyphosate Tolerant Bacteria to Perform Microbe-Assisted Phytoremediation
Source: Front Microbiol. 2021 Jan 14;11:598507. doi: 10.3389/fmicb.2020.598507 (PMC7840833; doi:10.3389/fmicb.2020.598507)
Supplement: Supplementary file 1 [file Data_Sheet_1.docx]

Supplementary Material


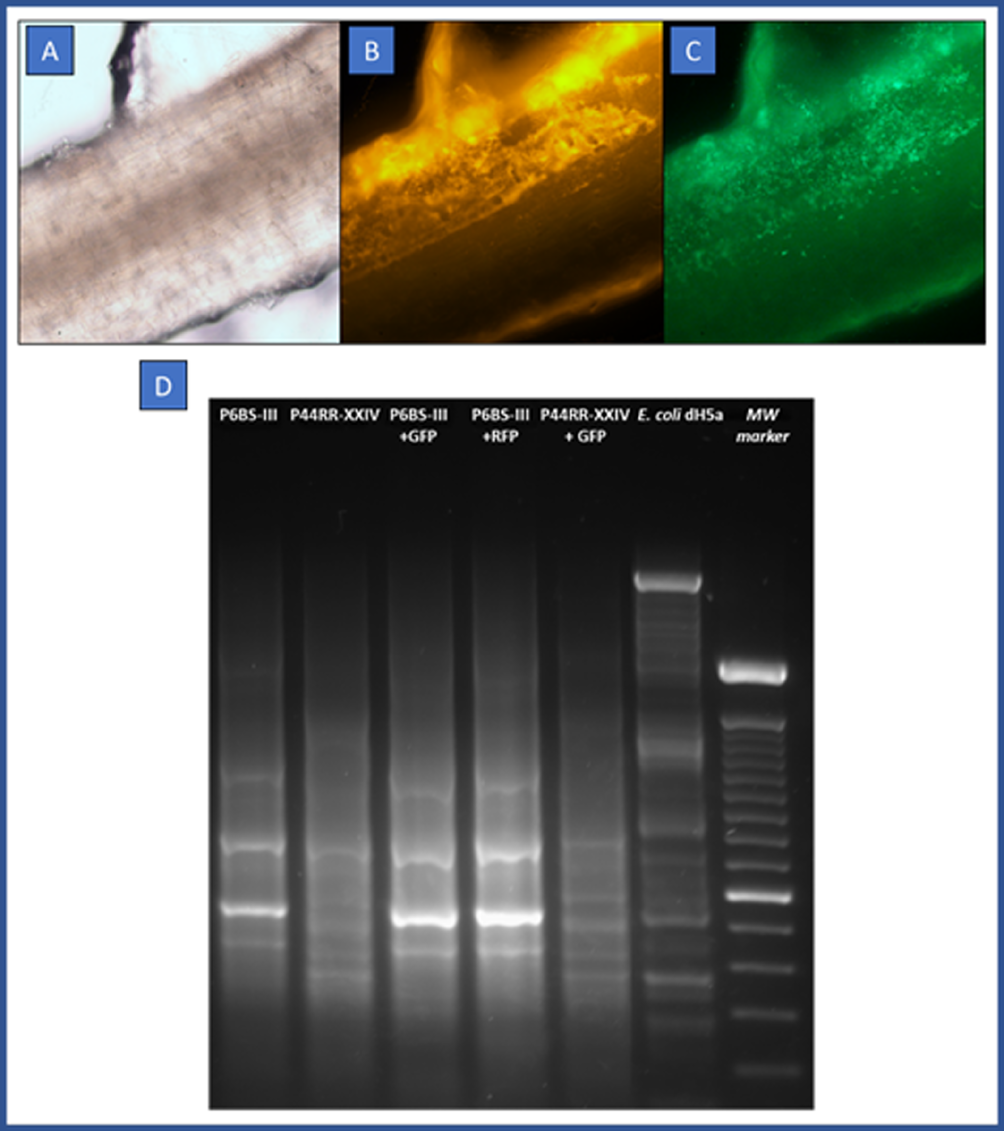


**Supplementary Figure 1**. Confirmation of positive conjugation in the bacterial strains. Microscopy image of a root section of *L. corniculatus* co-inoculated with *Rhizobium* sp. P44RR-XXIV GFP+ and O. *haematophilum* P6BS-III mCherry+ without fluorescence excitation (A). The same microscope field with excitation light 542/27, where *O. haematophilum* P6BS-III mCherry+ cells attached to the root can be seen (B). Same microscope field, with excitation light 480/40, where *Rhizobium* sp. P44RR-XXIV GFP+ cells attached to the root can be seen (C). BOX-PCR showing the different band patterns produced by the wild-type strains and transconjugants (D).


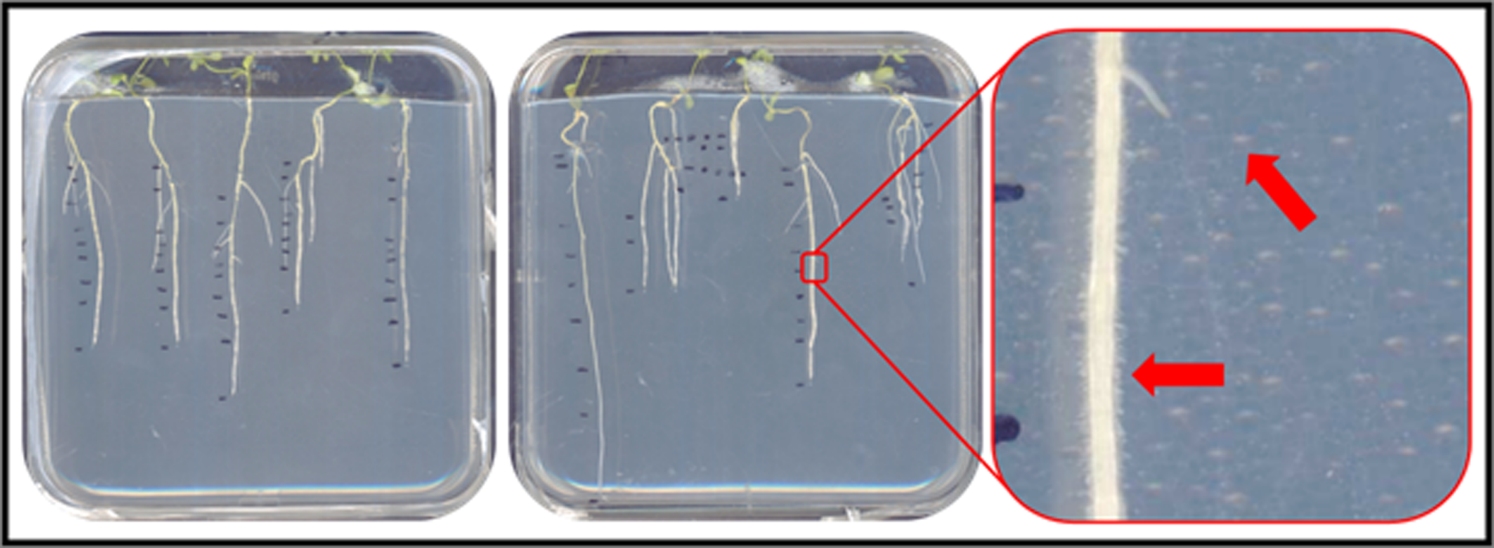


**Supplementary Figure 2**. Vertical agar plates with bacteria. A plate with non-inoculated seedlings (Left). A plate inoculated with *O.* *haematophilum* P6BS-III (middle). The same experiment was performed with *Rhizobium* sp. P44RR-XXIV (data not shown). An enlargement of one of the roots (right). The red arrows indicate the hairy roots and the abundant microbial growth on the plate, appearing as whitish homogenous colonies.


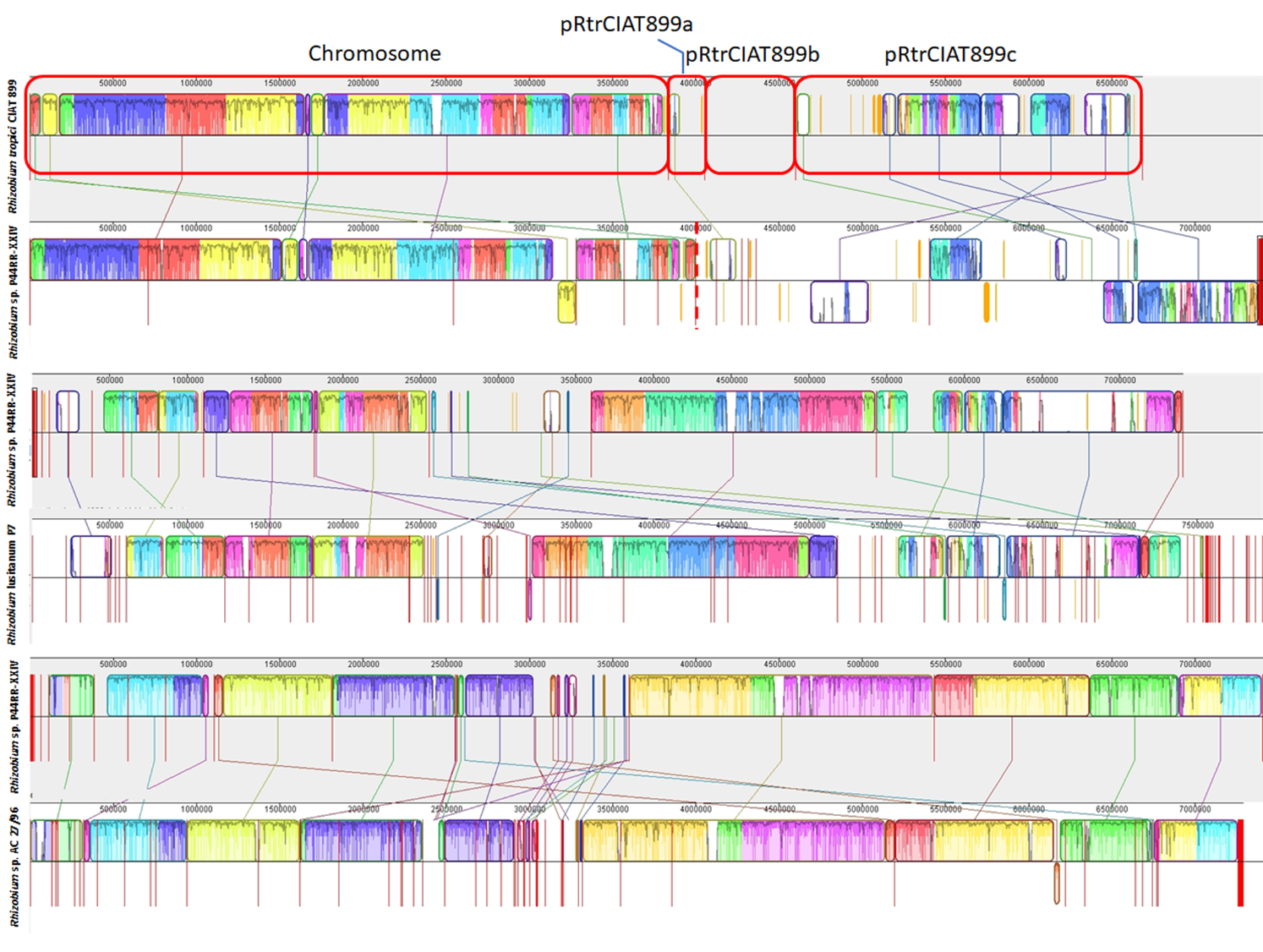


**Supplementary Figure 3**. Alignment and reordering of *Rhizobium* sp. P44RR-XXIV contigs was performed using the *Rhizobium tropici* CIAT 899 genome (top). P44RR-XXIV was then compared to the closest reference genome using 16s rRNA gene (middle), and the closest genome according to genome-to-genome methods comparison (down).


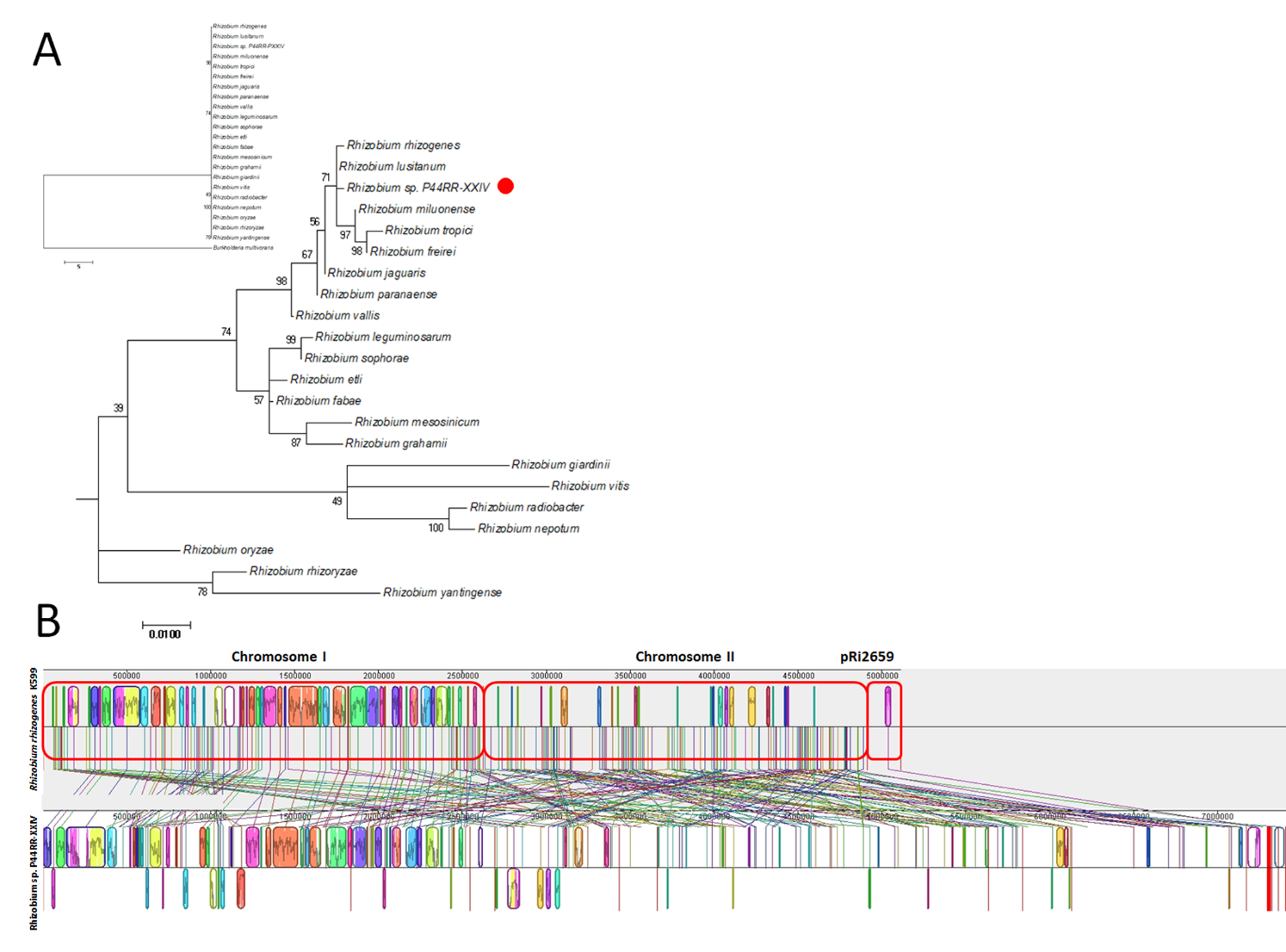


**Supplementary Figure 4**. Comparative study between *Rhizobium* sp. P44RR-XXIV and *R. rhizogenes* K599. A phylogenetic tree was performed using the 16s rRNA gene of the type strains of *Rhizobium* genus. Alignment was made using ClustalW algorithm, and phylogenetic analysis based on the maximum likelihood method was performed in MEGA7. Confidence levels for individual branches were determined by bootstrap analysis with 1000 replicates (A). The alignment of the whole genome sequence was performed together with the *R. rhizogenes* K599 using the Mauve software (B).


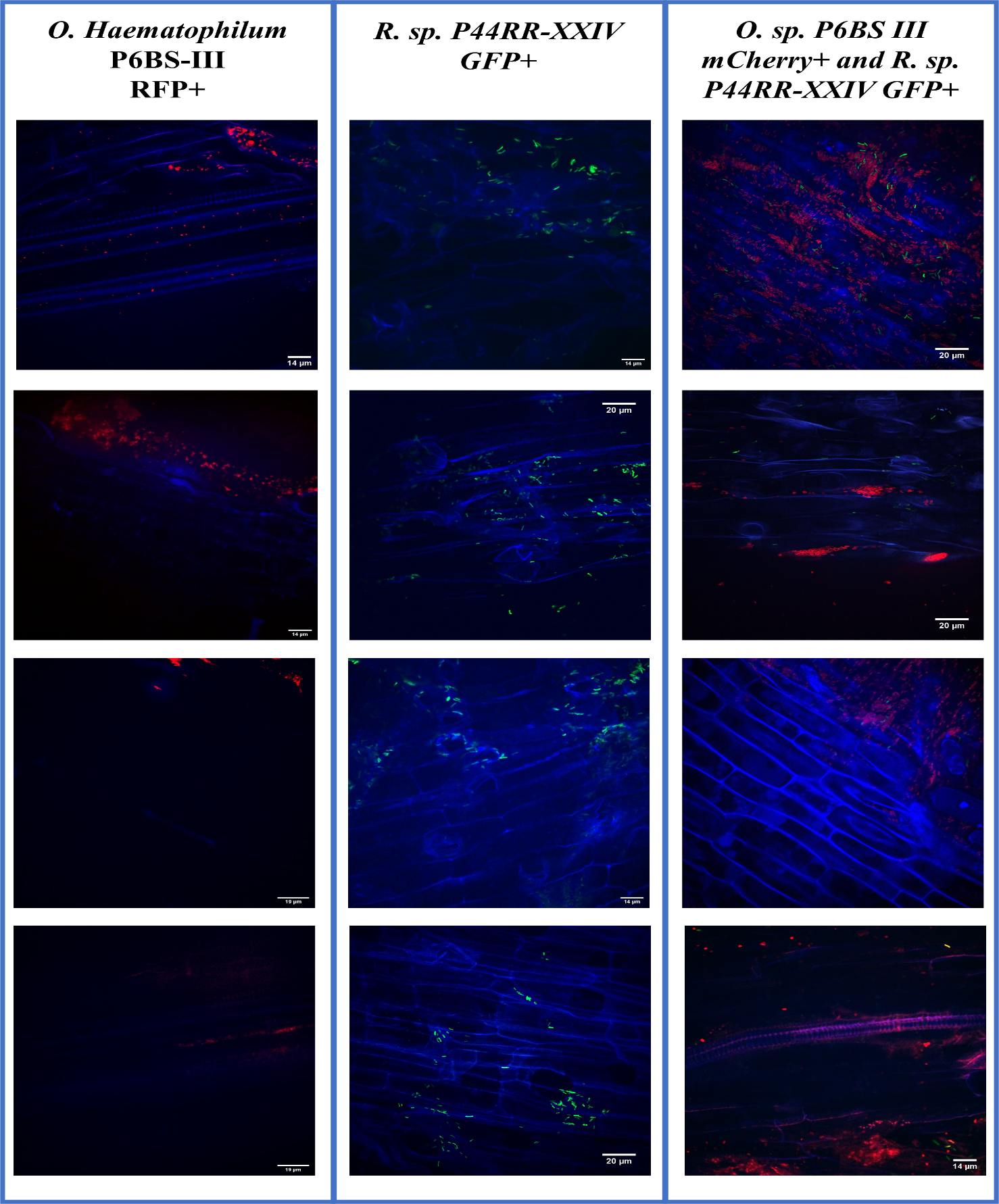


**Supplementary Figure 5**. Additional images of the confocal fluorescence microscopy. Lotus roots inoculated with the glyphosate degrading microorganisms. On the left, *O. haematophilum* P6BS-III RFP+. On the middle, *R*. sp. P44RR-XXIV GFP+. On the right*,* Lotus roots were inoculated with *O*. sp. P6BS III mCherry+ and *R*. sp. P44RR-XXIV GFP+.

| **Property** | | **Soil Plot** | | |
| --- | --- | --- | --- | --- |
|  |  | **Plot 1 and 2** | **Plot 3 and 4** |  |
| **Texture (%)** | Clay | 23 | 18 |  |
|  | Silt | 46 | 50 |  |
|  | Sand | 31 | 32 |  |
| **Element** | P mg Kg^-1^ | 6.36 | 8.6 |  |
|  | N % | 0.256 | 0.2 |  |
|  | K mEq 100g^-1^ | 1.1 | 1.1 |  |
|  | Ca mEq 100g^-1^ | 20 | 7.6 |  |
|  | Na mEq 100g^-1^ | 8.6 | 7.0 |  |
|  | MgmEq 100g^-1^ | 0.4 | 8.6 |  |
| **pH** |  | 8.3 | 6.8 |  |
| **Organic Matter (%)** |  | 5.9 | 4.5 |  |
| **CEC (mEq 100g^-1^)** |  | 6.7 | 7.4 |  |

**Supplementary Table 1**. Soil properties of the different agricultural plots sampled in this work. Plot 1 and 2 shared similar properties by proximity as well as plot 3 and 4.

| **Strain name** | **BioProject** | **Size (Mb)** | **GC %** | **ANIb (%)** | **ANIm (%)** | **GGDC (%)** |
| --- | --- | --- | --- | --- | --- | --- |
|  |  |  |  |  |  |  |
| *R. freirei* PRF 81 | PRJNA13459 | 7.08 | 59.96 | 83.48 | 86.62 | 29.2 |
| *Rhizobium* sp. AC27/96 | PRJNA319063 | 7.29 | 59.7 | 94.57 | 95.13 | 60.6 |
| *Rhizobium* sp. CF142 | PRJNA83051 | 7.46 | 60.2 | 75.16 | 84.23 | 21.5 |
| *Rhizobium* sp. YK2 | PRJNA324744 | 7.16 | 59.7 | 82.14 | 85.98 | 27.4 |
| *R. tropici* CIAT 899 | PRJNA42391 | 6.69 | 59.51 | 82.09 | 86.09 | 27.5 |
| *R. rhizogenes* NBRC 13257 | PRJNA224116 | 7.04 | 59.91 | 80.39 | 85.60 | 25.6 |
| *R. rhizogenes* ATCC 15834 | PRJNA240998 | 7.06 | 60 | 80.44 | 85.59 | 26.7 |
| *R. lusitanum* P7 | PRJEB14985 | 7.92 | 59.6 | 84.61 | 87.74 | 31.5 |
| *A. radiobacter* K84 | PRJNA13402 | 7.27 | 59.87 | 80.41 | 85.59 | 25.6 |
| *Rhizobium* sp. AP16 | PRJNA83035 | 6.50 | 60.21 | 80.45 | 85.57 | 25.6 |

**Supplementary Table 2**. Comparison between *Rhizobium* sp. P44RR-XXIV genome and closest genomes using different genome-to-genome comparison methods.
